# Supplementary material for: Influences of Semaphorin 3A Expression on Clinicopathological Features, Human Papillomavirus Status, and Prognosis in Oropharyngeal Carcinoma
Source: Microorganisms. 2020 Aug 22;8(9):1286. doi: 10.3390/microorganisms8091286 (PMC7565979; doi:10.3390/microorganisms8091286)
Supplement: Supplementary file 1 [file microorganisms-08-01286-s001.pdf]

Supplementary materials

Supplemental Figure S1

Comparison of different Kaplan-Meier curves for disease free survival for patients grouped by immunohistochemistry levels of SEMA3A.

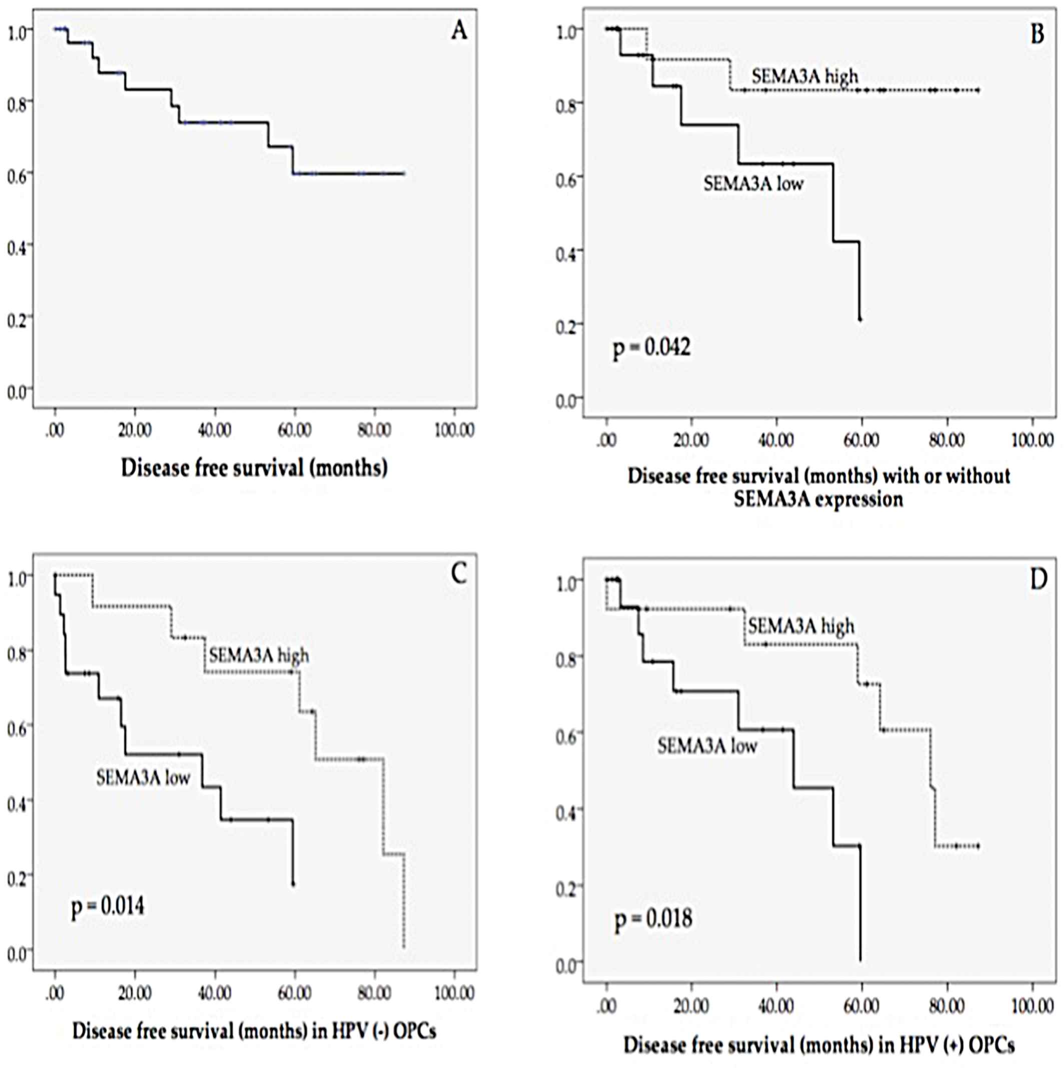

Supplemental Figure S1

(A). Kaplan-Meier curves for disease free survival (DFS) of the 32 OPC patients. (B). Kaplan-Meier curves for DFS for OPC patients with low and high levels of SEMA3A expression (P = 0.042). (C). Kaplan-Meier curves for DFS in patients who were HPV (+) with low and high levels of SEMA3A expression (P = 0.018). (D). Kaplan–Meier curves for DFS in patients who were HPV (-) with low and high levels of SEMA3A expression (P = 0.014).

Supplementary materials

Supplemental Table S1

Cox propotional hazard regression analysis of 32 OPC patients with overall survival

Variables	Univariate analysis		Multivariate analysis	
	HR (95% CI)	*p*	HR (95% CI)	*p*
Sex	1.379 (0.166-11.424)	0.76		NI
Age (years)	0.717 (0.143-3.588)	0.68		NI
Semaphorin 3A	0.192 (0.036-1.925)	0.05	0.307 (0.055-1.718)	0.179
Histological grade (poorly, moderately, well)	1.192 (0.504-2.815)	0.68		NI
Subsite (lateral, frontal, upper)	0.47 (0.072-3.054)	0.42		NI
TNM stage (I-II, III-IV)	1.696 (0.402-7.152)	0.47		NI
Tumor stage (T1-2, T3-4)	2.226 (0.549-9.024)	0.26		NI
Lymph node metastasis (negative, positive)	0.184 (0.036- 0.929)	0.04	0.275 (0.05-1.503)	0.136
HPV (negative, positive)	0.629 (0.15-2.640)	0.52		NI
Smoking	0.355 (0.067-1.881)	0.22		NI
Alcohol	1.776 (0.349-9.037)	0.48		NI

Factors with *p* value greater than 0.05 in the univariate models were not included (NI) in the multivariate analysis

P-value < 0.05 was considered statistically significant

Supplemental Table S1

A univariate Cox regression analysis did not show a significant association of age (≥50), sex, histological grade, subsite, tumor status, stage, HPV, smoking and drinking. Univariate Cox regression analysis showed that lymphonode metastasis and SEMA3A expression represented significant hazards.

Supplementary materials

Supplemental Table S2

Cox propotional hazard regression analysis of 32 OPC patients with disease free survival

Variables	Univariate analysis	
	**HR (95% CI)**	***p***
Sex	1.575 (0.191-12.959)	0.673
Age (years)	0.673 (0.135-3.366)	0.63
Semaphorin 3A	0.21 (0.41-1.078)	0.062
Histological grade (poorly, moderately, well)	1.223 (0.522-2.866)	0.643
Subsite (lateral, frontal, upper)	0.434 (0.064-2.963)	0.395
TNM stage (I-II, III-IV)	1.787 (0.425-7.504)	0.428
Tumor stage (T1-2, T3-4)	2.507 (0.615-10.228)	0.2
Lymph node metastasis (negative, positive)	0.147 (0.027- 0.792)	0.026
HPV (negative, positive)	0.635 (0.151-2.666)	0.535
Smoking	0.502 (0.108-2.329)	0.378
Alcohol	1.866 (0.366-9.498)	0.453

P-value < 0.05 was considered statistically significant

Supplemental Table S2

A univariate Cox regression analysis did not show a significant association of age (≥50), sex, SEMA3A, histological grade, subsite, tumor status, lymph node metastasis, stage, HPV, smoking and drinking.
